# Influence of life stress, 5-HTTLPR genotype, and *SLC6A4* methylation on gene expression and stress response in healthy Caucasian males

**DOI:** 10.1186/s13587-015-0017-x

**Published:** 2015-05-14

**Authors:** Elif A Duman, Turhan Canli

**Affiliations:** Integrative Neuroscience, Department of Psychology, Stony Brook University, Stony Brook, NY 11794-2500 USA; Department of Radiology, Stony Brook University, Stony Brook, NY 11794 USA; Program in Neuroscience, Stony Brook University, Stony Brook, NY 11794 USA; Program in Genetics, Stony Brook University, Stony Brook, NY 11794 USA; Department of Psychology, Bogazici University, Bebek, 34342 Istanbul, Turkey; Center for Life Sciences and Technologies, Bogazici University, Bebek, 34342 Istanbul, Turkey

**Keywords:** Early life stress, Serotonin transporter, Glucocorticoid receptor, Gene expression, DNA methylation, Hypothalamic-pituitary-adrenal axis, Depression

## Abstract

**Background:**

Previous research reported that individual differences in the stress response were moderated by an interaction between individuals’ life stress experience and the serotonin transporter-linked polymorphic region (5-HTTLPR), a common polymorphism located in the promoter region of the serotonin transporter gene (*SLC6A4*). Furthermore, this work suggested that individual differences in *SLC6A4* DNA methylation could be one underlying mechanism by which stressful life events might regulate gene expression. The aim of this study was to understand the relation between early and recent life stress experiences, 5-HTTLPR genotype, and *SLC6A4* methylation. In addition, we aimed to address how these factors influence gene expression and cortisol response to an acute psychosocial stressor, operationalized as the Trier Social Stress Test (TSST). In a sample of 105 Caucasian males, we collected early and recent life stress measures and blood samples to determine 5-HTTLPR genotype and *SLC6A4* methylation. Furthermore, 71 of these participants provided blood and saliva samples before and after the TSST to measure changes in *SLC6A4* and *NR3C1* gene expression and cortisol response.

**Results:**

Compared to S-group individuals, LL individuals responded with increased *SLC6A4* mRNA levels to the TSST (*t*(66) = 3.71, *P* < .001) and also showed increased global methylation as a function of ELS (*r* (32) = .45, *P* = .008) and chronic stress (*r* (32) = .44, *P* = .010). Compared to LL individuals, S-group individuals showed reduced *SLC6A4* mRNA levels (*r* (41) = −.31, *P* = .042) and increased F3 methylation (*r* (67) = .30, *P* = .015) as a function of ELS; as well as increased F1 methylation as a function of chronic stress and recent depressive symptoms (*r* = .41, *P* < .01), which correlated positively with *NR3C1* expression (*r* (42) = .31, *P* = .040).

**Conclusions:**

Both early and recent life stress alter DNA methylation as a function of 5-HTTLPR genotype. Some of these changes are also reflected in gene expression and cortisol response, differentially affecting individuals’ stress response in a manner that may confer susceptibility or resilience for psychopathology upon experiencing stressful life events.

**Electronic supplementary material:**

The online version of this article (doi:10.1186/s13587-015-0017-x) contains supplementary material, which is available to authorized users.

## Background

Studies of gene-by-environment interactions (GxE) have begun to reveal important clues regarding the etiology of depression. Much of this research enterprise has been devoted to the serotonin transporter gene (*SLC6A4*) and its interaction with stressful life events (SLEs). Serotonin (5-hydroxytryptamine, 5-HT) is an important neurotransmitter regulating the hypothalamic-pituitary-adrenal axis (HPA) stress response [1,2] and has been implicated in various mood disorders such as depression. The serotonin transporter is responsible for the reuptake of excess serotonin in the synaptic cleft and is commonly targeted by a class of antidepressants known as selective serotonin reuptake inhibitors. A common variant (polymorphism) located in the promoter region of the gene (*SLC6A4*) encoding the transporter, the serotonin transporter-linked polymorphic region (5-HTTLPR), has been widely studied with regard to individual differences in trait neuroticism, anxiety, and depression. The 5-HTTLPR is characterized by a short (S) and a long (L) allele that differ in transcriptional efficiencies, with the S allele being less active than the L allele [3,4]. In addition to the 5-HTTLPR, an A/G single nucleotide polymorphism (SNP; rs25531) located within 5-HTTLPR is suggested to alter the transcriptional efficiency of the L allele, such that the L_G_ allele is considered functionally similar to the S allele [5].

A seminal longitudinal gene-by-environment (GxE) study by Caspi and colleagues reported that the S allele moderated increased risk for depression associated with childhood maltreatment [6]. Subsequent work has produced conflicting results [7-10], which may be due, in part, to variation across studies in the selection of the type and timing of stressful life events (SLEs), sample size, and assessment of depression [10-13]. With regard to SLEs, some studies have considered early life stress (ELS) such as childhood maltreatment, whereas others have focused on recent SLEs. Indeed, it now appears that the timing of SLEs is a critical variable in revealing an interaction with 5-HTTLPR genotype: the most recent, large-scale meta-analysis by Karg *et al*. [10] specifically investigated the effect of stressor type and discovered that 5-HTTLPR strongly moderated the relationship between depression and ELS, specifically childhood maltreatment.

Studies investigating the sequelae of ELS at the molecular level suggest that these effects may be stable and persist through adulthood, such as alterations in the expression of *SLC6A4* [14-17] but also see [18]. One of these studies reported an additive effect of ELS and 5-HTTLPR genotype, such that S carriers with ELS exhibited the lowest level of baseline *SLC6A4* expression [16]. Given known interactions between serotonergic and glucocorticoid systems [19], ELS-mediated regulation of *SLC6A4* and its effect on the glucocorticoid receptor (NR3C1) could be one mechanism by which ELS can affect the HPA response. Activity of HPA is regulated both by negative feedback of cortisol by binding to NR3C1 and also through the action of neurotransmitters, such as serotonin, in specific brain regions [20]. Indeed, one recent study in rats reported that ELS and a polymorphism in *SLC6A4* leads to altered hippocampal *NR3C1* expression and cortisol release [21], suggesting that 5-HTTLPR may alter HPA activity through its effects on *NR3C1* regulation.

Recent studies have begun to study GxE interactions using laboratory-based social stress tasks, such as the Trier Social Stress Test (TSST) [22]; or derivations of the TSST. For example, Alexander *et al*. [23] reported a significant interaction between SLEs and 5-HTTLPR genotype in healthy males such that S carriers with a high number of SLEs exhibited the highest cortisol response. Mueller *et al*. [24] reported the same interaction in young adults (but not in children or older adults) for SLEs during the first 5 years of life, which was associated with increased cortisol responses in S-allele carriers but decreased cortisol responses in homozygous L-allele carriers. These studies, along with other neuroimaging (such as [25]) and behavioral (such as [26]) studies suggest that 5-HTTLPR genotype moderates stress reactivity and vulnerability to psychopathology in interaction with environmental variables.

Other work has begun to address the underlying molecular mechanisms of these GxE interactions, with a particular focus on epigenetic changes [27]. The first epigenetic study investigating the impact of ELS was conducted in rats [28], focusing on DNA methylation, which involves the addition of a methyl group to a segment of DNA containing a CpG dinucleotide. DNA methylation can alter gene expression when it occurs across long sections of CpG-rich regions (so-called ‘CpG islands’) but also when it occurs at specific sites, such as binding sites for specific transcription factors (TF) [29]. Researchers showed that ELS, operationalized as poor maternal care with low levels of licking and grooming of pups, was associated with increased DNA methylation at a particular CpG site in the hippocampal *NR3C1*, which was associated with lower gene expression and with higher HPA activation in response to stress in adulthood [28]. Subsequent studies provided further evidence for a similar methylation pattern in the postmortem hippocampi of suicide victims exposed to childhood abuse [30], in the cord blood of infants with depressed mothers during pregnancy [31], and in the blood of adults exposed to childhood maltreatment [32]. The similarity of results across the range of species and tissues studied suggests that DNA methylation in *NR3C1* may be a highly conserved and ubiquitous mechanism by which life stress may alter gene expression.

Other studies examined DNA methylation in *SLC6A4*, focusing on a CpG island in the promoter region of this gene, spanning an untranslated exon [33]. Several studies in humans and in non-human primates reported associations between ELS and DNA methylation across the CpG island or at specific CpG sites [34-39]. In addition, some studies reported associations between methylation of the whole CpG island (or sections of it) and gene expression [16,37,40], whereas others reported reduced gene expression as a result of *in vitro* methylation of certain CpG sites [41,42]. 5-HTTLPR genotype appears to further differentiate *SLC6A4* methylation, as some studies reported increased methylation in S carriers [33,37] and suggested that methylation of the S allele may exacerbate the impact of ELS [34] (although some reported the reverse pattern in relation to unresolved trauma [43]).

One recent study examined the association between stressful life experiences, *SLC6A4* methylation, and individuals’ cortisol response to during a modified form of the Trier Social Stress Test, TSST [38]. The study was based on 28 monozygotic twin pairs who were discordant for childhood bullying victimization [38] and reported that bullying victimization was associated with increased *SLC6A4* methylation and a blunted cortisol response to the TSST. The study did not address the putative moderating role of 5-HTTLPR genotype or the effect on *SLC6A4* mRNA gene expression. These questions were addressed across a set of two other studies. The first study was conducted in a population sample of 133 healthy young adults and reported an additive effect of early life stress (in the form of either prenatal stress or childhood maltreatment) and presence of the 5-HTTLPR S allele on reducing baseline *SLC6A4* mRNA gene expression [16]. These investigators also examined *SLC6A4* methylation and concluded that the observed differences in mRNA expression were unlikely to be mediated by methylation within this gene’s CpG island. In a second, follow-up study with an enlarged sample of 200 healthy young adults, this group then used the TSST to show that *SLC6A4* methylation moderated the association between 5-HTTLPR genotype and cortisol response to the TSST such that S carriers with low *SLC6A4* methylation had higher cortisol responses than LL homozygotes [44]. There were no differences in cortisol response by 5-HTTLPR genotype in the high *SLC6A4* methylation group. However, authors did not report any associations between *SLC6A4* methylation and life stress.

In light of the fact that no single prior study combined all of the putative elements of a molecular GxE interaction, the aim of the current study was to first investigate the interaction between ELS and 5-HTTLPR on *SLC6A4* methylation and its association with *SLC6A4* and *NR3C1* expression and cortisol response following the TSST. In addition, we investigated whether chronic stress and recent depressive symptoms would be associated with *SLC6A4* methylation, gene expression, and cortisol response as a function of 5-HTTLPR genotype, given that several studies reported correlations between depressive symptoms, antidepressant response, and methylation of different CpG sites in the *SLC6A4* CpG island [36,40,45,46].

## Methods

### Participants

Participants were 105 Caucasian males aged 18 to 77 (*M* = 28.51, SD = 13.82) who were recruited from Stony Brook University and surrounding communities through flyers, newspaper, and online advertisements. Participants were screened by phone for eligibility. All participants reported no prior diagnosis of psychological disorders or use of any related medication. Details of other exclusion criteria are given in Additional file 1. Measures of early life and chronic stress, 5-HTTLPR genotype, and DNA methylation (see below) were available from all of these participants. A subset (*N* = 71) participated in the TSST (*M*_age_ = 29.79, SD_age_ = 15.24). Additional data on recent depressive symptoms, gene expression, and cortisol response to the TSST were available from these participants. The study was approved by the Stony Brook University Institutional Review Board, and participants provided written consents prior to participation in the experimental sessions. At the end of each session, participants were debriefed orally and in writing and compensated with $100 plus reimbursement for any public transportation costs.

### Experimental sessions

To standardize biological measures under the influence of diurnal variation, all experimental sessions started between 12:00 and 14:00 h. Participants were instructed to refrain from eating, drinking (other than water), and exercise for at least 1 h before their arrival. The total procedure, which took about 4 h, included consenting, completion of questionnaires, the TSST, a life event interview, and debriefing. Participants also provided blood samples for genotyping and DNA methylation analyses, one at the beginning of the session (45 min before the TSST) and one at the end (105 min after the TSST). Cortisol levels were assessed using saliva samples collected at nine different time points throughout the session.

### Assessment of early life stress

Early life stress was assessed with the Childhood Trauma Questionnaire (CTQ) [47], which is a commonly used measure of childhood maltreatment consisting of 28 items with subscales of physical, sexual, and emotional abuse and physical and emotional neglect. Each subscale consists of five items, plus three items that serve to control for denial of maltreatment. The items are rated on a 5-point Likert scale (1 to 5), with higher scores indicating higher levels of maltreatment. Scores are added up to calculate the CTQ total score, which can range from 25 to 125.

### Assessment of chronic stress and recent depressive symptoms

Chronic stress for the last 3 months was assessed with the Trier Inventory of Chronic Stress (TICS) [48,49]. TICS is a 12-item self-report measure on the frequency of behaviors related to chronic stress, such as ‘I worry that I will not be able to fulfill my tasks’ and ‘I experience having too much to do.’ Each item is rated from 0 (never) to 4 (very often), and items are added up to calculate the total chronic stress score, which can range from 0 to 48.

Participants performing the TSST also completed the Beck Depression Inventory II BDI-II [50]; for assessing recent depressive symptoms. The BDI-II is a 21-item self-report measure of recent depressive symptoms (last 2 weeks) such as sadness, hopelessness, and self-blame. Each item is rated on a scale from 0 to 3, and scores can range from 0 to 63. Higher scores indicate higher depressive symptoms.

### Assessment of cortisol levels and stress reactivity

For the assessment of cortisol levels in response to the TSST, participants’ saliva samples were collected using salivettes (Sarstedt, Rommelsdorf, Germany). Forty-five minutes after the first blood draw and just prior to the beginning of the TSST, participants provided baseline saliva samples and were then taken to the TSST room. The TSST was performed as described in Kirschbaum *et al*. [22]. Briefly, the task consisted of a preparation phase (5 min), which was followed by a public speech (5 min) on why the participant would be the best candidate for his or her dream job and a backward-counting task (5 min). The task took place in front of a two-person committee that provided no verbal or non-verbal feedback. The active committee member, who gave instructions to the subject during the TSST, was always of the opposite sex (female); the inactive committee member, who did not communicate with the participant, was always of the same sex (male) as the participant. After the TSST, participants returned to the initial testing room and provided a second saliva sample right after the TSST and filled out an 8-item Visual Analog Scale (VAS) assessing the their experience of the TSST, such as finding it stressful, threatening, or challenging. Additional saliva samples were collected at 10, 20, 30, 45, 60, 90, and 105 min after the TSST. The saliva samples were stored at −20°C immediately after the session until being shipped to Brandeis University, Boston, for the analysis of cortisol concentration. Each sample was assayed in duplicates using a commercially available chemiluminescence immunoassay (RE62019) with a sensitivity of 0.16 ng/ml (IBL International, Toronto, ON, Canada). Inter- and intra-assay coefficients of variation were less than 7% and 4%, respectively. Cortisol peak increase was assessed as the difference between the peak cortisol level after the TSST and the baseline as used in prior studies [23,24]. For all participants, the highest response after the TSST was observed within 10 to 20 min after the TSST. We used peak response, rather than area under the curve, as a measure of cortisol reactivity, because the former is potentially more closely associated with changes in gene expression whereas the latter may be more closely associated with the overall hormonal output [51].

### Processing blood samples

In order to start with a uniform group of cells, peripheral blood mononuclear cells (PBMCs) were isolated from blood immediately after the blood draw, using Leucosep® tubes (Greiner Bio-One Inc., Monroe, NC, USA) and Ficoll-Paque (GE Healthcare, Pittsburgh, PA, USA) separation medium according to manufacturer’s protocol. The isolated PBMC pellets were stored at −80°C for subsequent DNA and RNA extraction procedures.

DNA and RNA extractions from PBMC pellets were carried out by the AllPrep DNA/RNA/Protein Mini kit (Qiagen, Valencia, CA, USA) according to manufacturer’s instructions. Quantity and quality of the DNA and RNA were assessed through NanoDrop ND-1000 (Thermo Scientific, Wilmington, DE, USA), DNA samples were stored at −20°C, and RNA samples were stored at −80°C.

### Genotyping of 5-HTTLPR and rs25531

5-HTTLPR genotype was determined through PCR amplification of 25 ng DNA at an annealing temperature of 67.5°C using primers employed in previous work [5]. A random subset of 24 samples was processed twice by a technician blind to the initial results to establish test-retest reliability, which was 100%. As a result of genotyping, individuals were genotyped as S/S, S/L, or L/L.

For genotyping the A/G SNP (rs25531), 6 μl of the 5-HTTLPR PCR products were digested with 5 Units of *Hpa*II restriction enzyme (New England Biolabs, Ipswich, MA, USA) for 3 h at 37°C. As a result, individuals were genotyped as S/S, S/L_A_, S/L_G_, L_A_/L_A_, L_A_/L_G_, and L_G_/L_G_. Given that expression of L_G_ allele was suggested to be similar to the S allele [52], the triallelic (S, L_A_, L_G_) classification scheme grouped S/L_G_ and L_G_/L_G_ individuals as ‘S/S’ and L_A_/L_G_ individuals as ‘L/S’. Genotype distributions were in Hardy-Weinberg equilibrium according to both biallelic and triallelic classification schemes (*P* > .05).

### DNA methylation analyses

For the analysis of DNA methylation, 500 ng DNA from each participant at baseline was bisulfite treated by using the Epitect Bisulfite kit (Qiagen, CA) according to manufacturer’s instructions and stored at −20°C until used in methylation analyses. In addition, in all methylation analyses, 500 ng unmethylated (0%) and fully-methylated (100%) human DNA samples (Zymo Research, Irvine, CA, USA) were bisulfite treated along with participants’ samples to be utilized as bisulfite conversion controls.

### Global DNA methylation

The methylation of Long Interspersed Nuclear Element-1 (*LINE-1*) was used as a measure of global methylation both for investigation of associations with ELS (similar to [39]) and for controlling for global methylation when investigating gene-specific methylation (similar to [31]). *LINE-1* methylation was quantified in duplicates by using the PyroMark Q96 CpG LINE-1 kit (Qiagen, CA) in a PyroMark Q96 MD system at the Stony Brook University Genomics Core Facility according to manufacturer’s protocol and with the commercial primers provided with the kit. Details of the procedure are given in Additional file 1.

### *SLC6A4* CpG island DNA methylation

**Figure 1:**
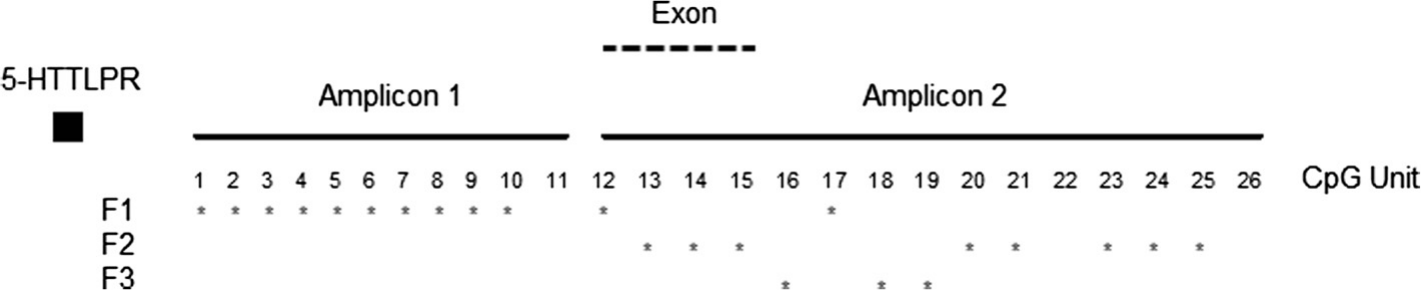
*SLC6A4* CpG island amplicons for DNA methylation analysis. Analyzed CpG Units are numbered from 1 to 26. The untranslated exon spans CpG Units 12 to 15 and 5-HTTLPR is located upstream of the CpG island. Asterisks show the CpG Units belonging to Factors 1, 2, and 3 (F1 to F3). F1 factor loadings ranged from .35 to .83, F2 factor loadings ranged from .37 to .76, and F3 factor loadings ranged from .73 to .87.

Methylation of the CpG island upstream of *SLC6A4* was quantified by the Sequenom Epityper MassArray system (San Diego, CA, USA). Two sets of primers were designed to amplify the 79 CpG sites in the CpG island in two amplicons similar to Philibert *et al*. [40] by using the Epityper software (Sequenom, CA). With this technique, methylation of *CpG Units* is analyzed, which can consist of one or more adjacent CpG sites. A total of 37 CpG Units were covered by the two amplicons, consisting of 79 CpG sites. All samples were run in triplicates. After preprocessing, methylation data from 26 CpG Units in amplicons 1 and 2 were included in all analysis (Figure 1). Details of the procedure, primer sequences, and data analysis are given in Additional file 1.

### Gene expression analyses

For each participant, two RNA samples were used for gene expression analyses, one 45 min before the TSST (baseline), and another 105 min after the TSST (response). Prior to quantification of gene expression, integrity of the RNA samples was assessed using the Agilent 2100 BioAnalyzer (Agilent Technologies, Santa Clara, CA, USA). RNA Integrity Numbers (RINs) of the samples were high (*M* = 7.94, SD = 1.32), and RINs of samples before and after the TSST did not differ significantly (*P* = .853). Afterwards, 1 μg of RNA from each time point was converted to cDNA using QuantiTect Reverse Transcription Kit according to the manufacturer’s protocol (Qiagen, CA). The cDNA samples were then diluted five times, and 1 μl of the diluted cDNA was used for the gene expression analysis of the candidate genes by quantitative PCR (qPCR), using the Qiagen SYBR Green PCR + UNG kit (Qiagen, CA) and gene-specific primers designed from the Roche Universal Probe Library website (http://www.roche-applied-science.com/sis/rtpcr/upl/ezhome.html). The qPCR reactions were carried out in triplicates in the Roche 480 LightCycler system (Roche Applied Science, Indianapolis, IN, USA) at an annealing temperature of 60°C.

To identify the best reference genes in PBMCs, the expression of six candidate reference genes was analyzed from five individuals’ RNA samples, obtained at baseline and response time points. This method identified *HPRT1* (hypoxanthine phosphoribosyltransferase 1) and *GAPDH* (glyceraldehyde 3-phosphate dehydrogenase) as the best reference genes in PBMCs. C_T_ values that were obtained by qPCR were then used to assess gene expression change between baseline and response samples, using the delta-delta-C_T_ method [53]. Changes in gene expression for each sample, normalized for reference genes, are shown as fold-change values, representing the fold change in *SLC6A4* and *NR3C1* after the TSST relative to baseline. Details of the qPCR analyses and primer sequences are given in Additional file 1.

### Statistical analysis

All statistical analyses were performed using SPSS for Windows version 16.0 (Chicago, IL, USA), with significance level set at *α* = .05. In order to assess whether the TSST successfully evoked a cortisol response, we used a repeated measures ANOVA for the nine saliva samples collected throughout the experiment. Prior to all analyses, cortisol data were tested for normal distribution by the Kolmogorov-Smirnov test. Due to violation of normality for samples at multiple time points (*P* < .05), log transformation was applied to all cortisol data. Due to the violation of sphericity (*P* < .05), Greenhouse-Geisser correction was applied.

To examine correlations between variables of interest, Pearson’s correlation coefficient (*r*) was used for the normally distributed variables, and Spearman’s rho coefficient (*rs*) was used for the non-normally distributed variables. Partial correlations were used as necessary to control for the effects of some variables such as age and *LINE-1* methylation.

In order to understand the methylation patterns across the CpG island and reduce the number of variables investigated, a factor analysis covering the 26 CpG Units across the island was conducted similar to Olsson *et al*. [41]. Kaiser-Meyer-Olkin measure of sampling adequacy (.834) and Bartlett’s test of sphericity (*P* < .001) suggested that factor analysis is suitable for the data set. As a result of the analysis, five factors emerged explaining 75% of the variance. However, since fewer than three variables were loaded onto the last two factors, only the first three factors were considered: Factor 1 (F1), Factor 2 (F2), and Factor 3 (F3). Percentage of the variance explained by F1, F2, and F3 were 37, 15, and 12, respectively. Loadings on these factors were such that F1 primarily included CpG Units at the beginning of the CpG island up to the start of the exon, whereas F2 included those towards the end of the island and F3 included a shorter region towards the end of the island (Figure 1).

## Results

### Participant characteristics and response to the TSST

The study included 105 Caucasian males aged 18 to 77 (*M*_age_ = 28.51, SD_age_ = 13.82). The distribution of 5-HTTLPR genotype groups is shown in Table 1. Furthermore, age did not vary as a function of 5-HTTLPR genotype (*t*(103) = 1.19, *P* = .238; S group *M* = 27.39, SD = 13.12; LL group *M* = 30.77, SD = 15.07). ELS, as measured by CTQ total scores, ranged from 25 to 66 (*M* = 34.83, SD = 9.57). Only two participants reported past sexual abuse (scoring 7 within a range of 5 to 25). Apart from sexual abuse, all CTQ subscale scores were significantly correlated with each other (*r =* .31 to .63) and with the CTQ total score (*r =* .67 to .86; *P* values ≤.001; individual correlation coefficients are reported in Additional file 1: Table S1). Chronic stress for the last 3 months, as measured by TICS total scores, ranged from 0 to 42 (*M* = 17.42, SD = 9.70). None of these measures differed as a function of 5-HTTLPR genotype (S *vs*. LL; *P* values >.860). Current depressive symptoms (BDI-II) of TSST participants ranged from 0 to 30 (*M* = 7.37, SD = 7.20) and did not differ as a function of 5-HTTLPR genotype (*P* = .646).

**Table 1:** **5-HTTLPR genotype distributions**

	**All**	**TSST participants only**
SS	24	15
SL	46	30
LL	35	26
Total	105	71

*Note.* Genotypes are given according to the triallelic classification.

**Figure 2:**
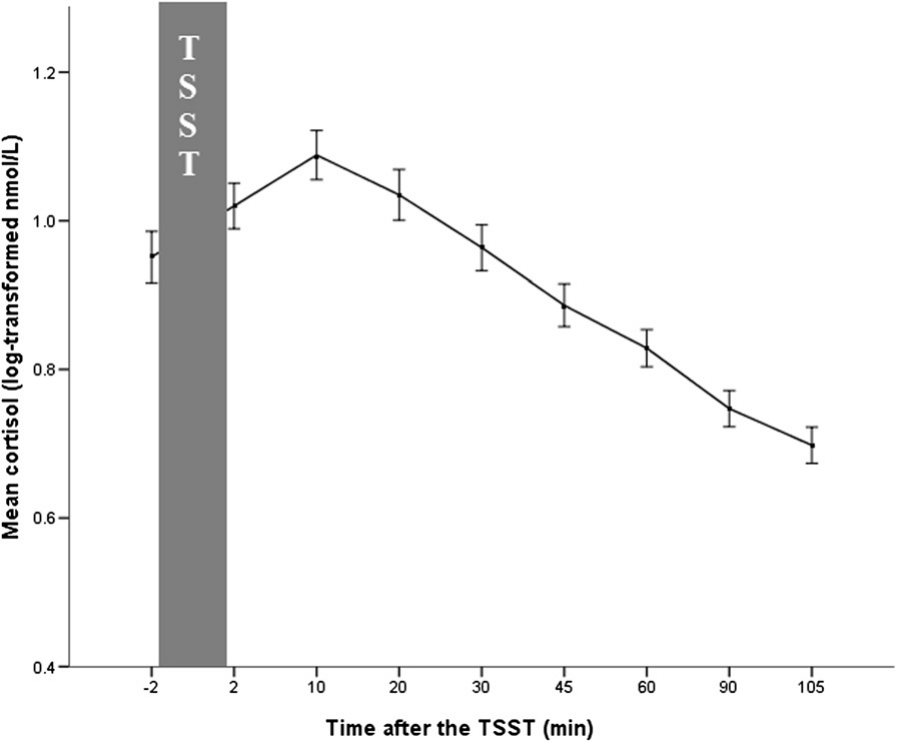
Cortisol response to the TSST. Cortisol levels (mean ± SE of mean) at different time points after the TSST.

A repeated measures ANOVA showed a significant cortisol increase to the TSST as shown in Figure 2 (*F*_2.69, 70_ = 61.41, *P <* .001, partial *η*^2^ = .47). 5-HTTLPR genotype was not associated with significant differences in the overall cortisol response (*P* = .758) or in baseline cortisol levels (*P* = .900).

### *SLC6A4* and *NR3C1* expression at baseline as a function of 5-HTTLPR genotype and early life stress

The first question we addressed was whether individuals differed in levels of serotonin transporter gene (*SLC6A4*) or glucocorticoid receptor gene (*NR3C1*) expression, as a function of either 5-HTTLPR genotype or early life stress, prior to any social stress exposure at baseline. We found no significant differences across any of our measures.

There were no significant differences in baseline *SLC6A4* expression as a function of 5-HTTLPR genotype (*t*(66) = −.79, *P* = .430; Additional file 2: Figure S1, panel A). There was also no significant correlation between baseline *SLC6A4* expression and ELS for the sample as a whole or as a function of 5-HTTLPR genotype (all *r* coefficients < .1, *P* values ≥ .539). The absence of any ELS effects was also noted when we converted measures of ELS from a continuous to a categorical variable: either by taking the median split of CTQ total scores (*F*_3, 63_ = .86, *P =* .358) or applying higher cut-off values used in prior studies to divide participants into ‘no-ELS’ or ‘ELS’ groups, *F*_3, 63_ = .69, *P =* .410 [similar to 16; detailed in Additional file 1]. There were also no effect of 5-HTTLPR and ELS interaction for both median split ELS scores (*F*_3, 63_ = .08, *P =* .772) or higher cut-off ELS scores (*F*_3, 63_ = .01, *P =* .910) on *SLC6A4* baseline expression.

There were no significant differences in baseline *NR3C1* expression as a function of 5-HTTLPR genotype (*t*(66) = .093, *P* = .926; Additional file 2: Figure S1, panel C). There was also no significant correlation between baseline *NR3C1* expression and ELS for the sample as a whole or as a function of 5-HTTLPR genotype (all *r* coefficients < .1, *P* values ≥ .454). Similar results were obtained when utilizing categorical ELS values and investigating their interaction with 5-HTTLPR genotype (all *P* values ≥ .307).

### *SLC6A4* and *NR3C1* expression in response to the TSST as a function of 5-HTTLPR genotype and early life stress

The next question we addressed was whether individuals differed in levels of serotonin transporter gene (*SLC6A4*) or glucocorticoid receptor gene (*NR3C1*) expression, as a function of either 5-HTTLPR genotype or early life stress, in response to the TSST. We found differential effects for *SLC6A4* but not *NR3C1.*

In response to the TSST, we found that LL individuals responded with increased *SLC6A4* mRNA expression to the TSST whereas S-group individuals’ expression values remained unchanged; LL individuals had significantly higher *SLC6A4* expression in response to the TSST than did S-group individuals (*t*(66) = 3.71, *P* < .001) (Additional file 2: Figure S1, panel B).

We also wanted to examine whether *SLC6A4* mRNA expression varied as a function of an interaction between 5-HTTLPR genotype and ELS. Although a formal interaction between 5-HTTLPR genotype and ELS was not significant (*F*_3, 63_ = .91, *P* = .343), we conducted exploratory analyses for each genotype separately, which showed that *SLC6A4* expression was inversely correlated with gene expression for S-group individuals (*r* (41) = −.31, *P* = .042) but not for LL individuals (*r* (23) = −.01, *P* = .967). Similar results were obtained when ELS was categorized as no-ELS *vs*. ELS (*F*_3, 63_ = 5.83, *P = .*001), such that LL individuals with ELS had the highest *SLC6A4* expression change, followed by LL with no-ELS, S carriers with no-ELS and S carriers with ELS. The results remained unchanged when a biallelic classification scheme was used.

*NR3C1* expression in response to the TSST did not differ as a function of 5-HTTLPR genotype (*t*(66) = −1.46, *P* = .149; Additional file 2: Figure S1, panel D). *NR3C1* expression in response to the TSST also did not differ as a function of ELS for the entire sample, nor for any of the subsamples as a function of 5-HTTLPR genotype (all *r* coefficients < .2, *P* values ≥ .237).

### Global DNA and *SLC6A4* methylation

Global methylation levels, as measured by *LINE-1* methylation, ranged from 71% to 80% (*M* = 74.77, SD = 2.38). There was a negative correlation between age and *LINE-1* methylation as expected [54]; *r*(103) = −.25, *P* = .011. There were no differences in *LINE-1* methylation as a function of 5-HTTLPR genotype (*P* = .699).

*SLC6A4* CpG island average methylation ranged from 7% to 14% (*M* = 8.85, SD = 1.67), increasing from the 5′ to the 3′ end; intercorrelations between CpG Units were higher in the 5′ end than the 3′ end as previously reported [40].

Average *SLC6A4* methylation correlated positively with age (*r*(103) = .33, *P* = .001), consistent with the literature suggesting increases in promoter methylation by age [55]. Controlling for age, *SLC6A4* average methylation was also correlated with *LINE-1* methylation (*r*(102) = .52, *P* < .001).

Age was positively correlated with F2 (*r*_s_ (103) = .34, *P* < .001) and F3 (*r*_s_ (103) = .40, *P* < .001) but not with F1 methylation (*r*_s_ (103) = −.02, *P* = .867). Controlling for age, *LINE-1* methylation was positively correlated with F1 (*r* (102) = .60, *P* < .001) and F2 (*r* (102) = .33, *P* = .001) methylation but negatively with F3 methylation (*r* (102) = −.24, *P* = .014).

### Early life stress: association with global DNA and *SLC6A4* methylation

In light of the previously discussed literature, we next addressed the question whether ELS is associated with either global DNA or *SLC6A4* methylation. There was no main effect and only a trend for a significant interaction between ELS and 5-HTTLPR (*F*_3, 67_ = 3.74, *p* = .056). An exploratory analysis for each genotype showed that ELS correlated positively with *LINE-1* methylation only in LL participants (*r* (32) = .45, *P* = .008), controlling for age. However, this correlation became non-significant once we controlled for chronic stress (*r* (31) = .27, *P* = .136).

There was no significant correlation between ELS and average *SLC6A4* methylation (*r* (103) = .09, *P* = .380), and results remained unchanged when controlling for age and *LINE-1* methylation. There was also no correlation between ELS and any of the three *SLC6A4* methylation factors, controlling for age and *LINE-1* methylation (*r* coefficients < .1, *P* values ≥ .090). However, when 5-HTTLPR genotype was taken into account (again controlling for age and *LINE-1* methylation), there was a significant interaction between 5-HTTLPR and ELS on F3 methylation (*F*_5, 99_ = 7.98, *P* = .006): ELS and F3 methylation correlated positively in S-group participants (*r* (67) = .30, *P* = .015) but not for LL participants (*r* (31) = −.28, *P* = .118). There were no significant correlations between ELS and F1 or F2 methylation by 5-HTTLPR genotype (Additional file 1: Table S3).

### *SLC6A4* methylation: association with *SLC6A4* and *NR3C1* expression and with cortisol release

Given that DNA methylation can regulate gene expression, we next addressed the question whether *SLC6A4* methylation was associated with differential gene expression of either *SLC6A4* or *NR3C1*. There was no significant correlation between *SLC6A4* methylation and *SLC6A4* expression overall, for any of the three factors, or as a function of 5-HTTLPR genotype (*r* coefficients < .2, *P* values ≥ .203).

There was also no significant correlation between *SLC6A4* methylation and *NR3C1* expression overall (*r* (68) = .21, *P* = .082), nor a significant interaction (F3, 66 = 2.21, *P* = .142). Exploratory analyses showed a significant correlation between overall *SLC6A4* methylation and *NR3C1* expression for S-group participants only (*r* (42) = .34, *P* = .023). In particular, there was also a correlation between F1 methylation and *NR3C1* expression in S-group participants only, (*r* (42) = .31, *P* = .040). Neither F2 nor F3 methylation were correlated with *NR3C1* expression for any of the genotype groups (Table 2).

**Table 2:** **Correlations between** ***SLC6A4*** **methylation and** ***NR3C1*** **expression**

***NR3C1*** **expression**	**F1 methylation**		**F2 methylation**		**F3 methylation**		**Average methylation**	
	**S**	**LL**	**S**	**LL**	**S**	**LL**	**S**	**LL**
Baseline	.21	.35	.14	.34	−.05	−.12	.09	.16
Response	.31*	.20	−.03	.06	.14	−.37	.34*	−.02

*Note.* ^*^ *P* < .05. Significant correlations are shown in italics.

To further assess the functional significance of these observations, we conducted additional correlational analyses with participants’ cortisol responses. There was no significant correlation between *NR3C1* expression and cortisol response in any of the genotype groups; nor was there a significant correlation between *SLC6A4* F1 methylation and cortisol response in any of the genotype groups. Only when *SLC6A4* F1 methylation results were further subdivided into tertiles did some differential effects emerge genotype (Additional file 3: Figure S2).

We also addressed the functional significance of *SLC6A4* methylation in regard to cortisol release in response to the TSST. There were no significant correlations between cortisol release and *SLC6A4* methylation (overall or for F1 to F3) for the sample overall or as a function of 5-HTTLPR genotype (all *r* coefficients < .2, *P* > .110).

### Chronic stress, recent depressive symptoms: association with global DNA and *SLC6A4* methylation

As discussed in the introduction, a meta-analysis of GxE interactions differentiated between early life stress and other stressors. Having examined ELS in the previous sections, we now turned to the association of chronic stress and recent depressive symptoms with global DNA and *SLC6A4* methylation.

Chronic stress (controlling for age) correlated significantly with global DNA methylation, as measured by *LINE-1* methylation, for the overall sample (*r* (102) = .23, *P* = .019). This correlation was driven by LL participants (*r* (32) = .44, *P* = .010) but not by S-group carriers (*r* (67) = .15, *P* = .219). This effect remained unchanged when controlling for ELS, as well. Recent depressive symptoms did not correlate significantly with *LINE-1* methylation for the overall sample (*r* (68) = .19, *P* = .111), nor as a function of 5-HTTLPR genotype (S carriers: *r* (42) = .19, *P* = .209; LL: *r* (23) = .22, *P* = .284).

Chronic stress (controlling for age and *LINE-1* methylation) did not correlate significantly with overall *SLC6A4* methylation for the overall sample, nor as a function of 5-HTTLPR genotype (Table 3). Recent depressive symptoms correlated only marginally with overall *SLC6A4* methylation (*r*(67) = .22, *P* = .064; Table 3). When we investigated the association of chronic stress and recent depressive symptoms for each *SLC6A4* methylation factor separately, there was a significant interaction between 5-HTTLPR and chronic stress (*F*_5, 99_ = 4.01, *P* = .048) but not for recent depressive symptoms (*F*_5, 66_ = 1.17, *P* = .283) on F1 methylation. A follow-up analysis by genotype found that the correlations were driven by the S group (*r* = .41, *P* < .01) but not LL participants (*r* = -.16, *P* > .519; Table 3). The results remained unchanged when controlling for ELS as well.

**Table 3:** **Partial correlations between SLC6A4 methylation, chronic stress, and recent depressive symptoms**

	***SLC6A4*** **methylation**			
	**Average**	**F1**	**F2**	**F3**
All				
Recent depressive symptoms	.22	.30*	.08	−.17
Chronic stress	.05	.20*	−.04	−.16
S group				
Recent depressive symptoms	.28	.41**	.08	−.16
Chronic stress	.20	.41**	.01	−.25
LL				
Recent depressive symptoms	−.02	.14	−.11	−.23
Chronic stress	−.16	−.11	.04	−.10

*Note*. Partial correlations controlling for age and *LINE-1* methylation. ^*^ *P* < .05; ^**^ *P* < .01. Significant correlations are shown in italics.

A summary of the current study findings is given in Table 4.

**Table 4:** **Summary of the major study findings**

	**DNA methylation**	**Gene expression change in response to the TSST**
		*SLC6A4* expression: LL > S-group
Early life stress ↑	LINE-1 methylation: S-group ↔ LL ↑	*SLC6A4* expression: S-group ↓ LL ↔
	F3 methylation: S-group ↑ LL ↔	
Chronic stress ↑	LINE-1 methylation: S-group ↔ LL ↑	F1 methylation correl w/ *NR3C1* expression: S-group ↑ LL ↔
	F1 methylation: S-group ↑ LL ↔	

*Note*. Arrows pointing up or down indicate significantly increased or decreased methylation/expression, respectively. Sideway arrows indicate no significant change.

## Discussion

This study aimed to address mechanisms of GxE interactions shaping individual differences in social stress reactivity. Using the TSST as a well-validated social stress paradigm, we investigated the relationship between SLEs and genetic and epigenetic variations, as well as their downstream effects on gene expression and HPA activity. We focused in particular on *SLC6A4*, which has been shown to moderate the effects of stressful life events and HPA reactivity.

We began our analyses with an *in vivo* blood-based assessment of serotonin transporter (*SLC6A4*) or glucocorticoid receptor (*NR3C1*) mRNA expression at baseline, as a function of either 5-HTTLPR genotype or ELS. We found no significant differential gene expression at baseline as a function of genotype or life stress, nor an interaction between these two variables. One other group, focusing on *SLC6A4,* conducted a similar study and did report lower levels of *SLC6A4 mRNA* expression as a function of 5-HTTLPR genotype and ELS, as well as an additive effect of these two variables [16]. Our sample was about 20% smaller, and it is therefore possible that we lacked statistical power to detect a difference.

When we turned our attention to the dynamic regulation of *SLC6A4* expression following exposure to the TSST, we found evidence for a regulatory role of 5-HTTLPR genotype: LL homozygotes showed a 1.5-fold increase in gene expression following the stressor, whereas S-group individuals’ expression levels remained unchanged. This finding is consistent with Mueller *et al*.’s study showing increased responsiveness to the TSST in LL homozygotes (when collapsed across SLEs), as measured by cortisol activation [24]. This finding is also consistent with Glatz *et al*.’s study [56] that reported less expression of the S allele following glucocorticoid stimulation. One reason for the difference in dynamic gene expression as a function of 5-HTTLPR genotype may be related to structural features in the promoter region of *SLC6A4* (as determined by the PROMO software; [57]), which contains several transcription factor binding sites (for example, AP-2, GR-α, Sp1) that are important in the glucocorticoid system [3,56]. Another reason may be differential affinity for methylation-related proteins as a function of 5-HTTLPR genotype [58]. To our knowledge, previous human studies did not consider the effect of 5-HTTLPR genotype on stress-related dynamic changes in PBMC *SLC6A4* expression. Thus, replication of this result in larger samples would be important.

Although change in *SLC6A4* expression was not affected as a function of ELS *per se*, it was affected as a function of the interaction between ELS and 5-HTTLPR genotype: ELS further exacerbated the reduced rate of change in *SLC6A4* expression to the TSST in S carriers but not in LL homozygotes. This result is similar to a set of rhesus macaque studies, which reported that ELS was associated with decreased *SLC6A4* expression in response to a stressor [14,59]. However, these studies did not report a differential effect of 5-httlpr genotype, which may reflect lacking statistical power due to the low number of subjects, particularly of SS genotype. The life stress effects we observed were specific to ELS, because we did not find a correlation between recent stress events and *SLC6A4* expression, consistent with another recent study [16]. Thus, our data suggest a model by which ELS appears to differentially amplify the genotype effects on dynamic *SLC6A4* expression in S carriers, which may render them vulnerable for psychopathology following stress exposure.

ELS may alter gene expression through modifications in DNA methylation [33]. To address this question, we considered both global *LINE-1* and gene-specific *SLC6A4* methylation. Indeed, ELS (as well as chronic stress) correlated positively with *LINE-1* methylation but only in LL participants. These results are consistent with a recent study in LL macaques that also reported an association between animals’ stress response and global and *SLC6A4* methylation in individuals who had experienced ELS [39]. ELS and chronic stress may affect *LINE-1* methylation through a common pathway, because when we controlled for chronic stress, the correlation between ELS and *LINE-1* methylation became nonsignificant. Future work conducted with larger samples should replicate this finding and examine possible molecular mechanisms by which chronic stress and ELS may alter *LINE-1* methylation.

ELS did not correlate significantly with average *SLC6A4* methylation, nor with methylation of any of the three *SLC6A4* methylation factors (F1 to F3). However, ELS may be associated with site-specific *SLC6A4* methylation as a function of 5-HTTLPR genotype: ELS correlated positively with F3 (but not with F1 or F2) methylation but only in S-Group participants. This finding suggests that not all CpG regions within *SLC6A4* may be equally sensitive to ELS as a function of 5-HTTLPR genotype. Given our small sample, this conclusion remains speculative until replicated in a larger sample.

One way to assess whether methylation of a particular CpG region is functionally significant is to consider it in the context of gene expression. Here, we found no evidence that F3 methylation, as a function of ELS and 5-HTTLPR genotype, correlated significantly with *SLC6A4* mRNA expression. Indeed, there was no evidence for any relation between *SLC6A4* methylation and *SLC6A4* mRNA expression overall, for any of the three factors, or as a function of 5-HTTLPR genotype. This observation is consistent with another study that concluded that methylation of *SLC6A4* was unlikely to moderate its expression [16].

It is possible, however, that *SLC6A4* methylation may exert an effect through indirect pathways, by regulating *NR3C1* expression [21]. Indeed, we discovered that there was a significant correlation between overall *SLC6A4* (as well as F1) methylation and *NR3C1* expression but only for S-group participants. This association did not, however, produce a corresponding differential cortisol response as one might expect [21], casting some doubt on the functional significance of this association.

In addition to ELS, we also examined the association of chronic stress and current depressive symptoms with global and with *SLC6A4* methylation (albeit, the exclusion of participants with a history of, or current, depression restricted the range of observed depressive symptoms). We found that both chronic stress and recent depressive symptoms were positively correlated with F1 methylation (a region in the 5′ end of the *SLC6A4* CpG island) in S group but not LL participants. Importantly, these results remained significant when we controlled for age, *LINE-1* methylation, and ELS. Given that short allele carriers exposed to life stress are more likely to show depressive symptoms [6], these findings are in line with previous studies that associated higher *SLC6A4* methylation with depression [36,40,45]. Our F1 region overlaps with a region examined in a study of MZ twins that reported a positive association between BDI scores and methylation [45]. This F1 region also overlaps with one examined in a study of job burnout in nurses [60]: when adjusted for working environment (high *vs*. low stress), methylation of this region was positively associated with increased burnout. The study found no influence of 5-HTTLPR genotype, which may reflect limited statistical power due to sample size. Taken together, our results and these other studies suggest that the region overlapping with F1 of the *SLC6A4* CpG island may be particularly plastic and sensitive to the effects of chronic or recent (as opposed to early) stressors.

*SLC6A4* F1 methylation may exert its effects by regulating gene expression. Although there was no association between *SLC6A4* F1 methylation and *SLC6A4* expression, there was a link with *NR3C1* expression. This link may reflect underlying structural features of the F1 region, which contains multiple binding sites for glucocorticoid receptors as determined by the PROMO software [57]. The link between *SLC6A4* F1 methylation and *NR3C1* expression was genotype specific: F1 methylation was positively correlated with *NR3C1* expression in S group but not LL individuals. The functional significance of this observation remains tentative because there was no significant correlation between either *SLC6A4* F1 methylation or *NR3C1* expression, on the one hand, and cortisol response to the TSST, on the other. Exploratory data suggest that level of F1 methylation may be a moderating factor, but our sample was too small to make any conclusive statements (Additional file 3: Figure S2).

### Strengths and limitations

One of the strengths of this study is that our sample was homogeneous. We limited enrollment to Caucasians to minimize ethnic stratification. We enrolled only males to minimize the confounding effects of steroid hormones on HPA reactivity. We excluded individuals with a previous diagnosis of psychopathology and related medication use. These criteria are important considering their confounding effects on DNA methylation [46,61], brain activity, and physiology [62].

The homogeneity of our sample comes at the cost of limited generalizability, since our data cannot be extrapolated to non-Caucasians, women, or individuals with psychopathology. Furthermore, the exclusion of individuals with diagnosed psychopathology may have limited the number of study participants with high levels of ELS (*N* = 18). Thus, the results of our analyses involving ELS should be considered preliminary, particularly when this cohort is further divided by 5-HTTLPR genotype. We also did not correct for multiple testing due to the exploratory nature of the study. Therefore, we suggest that future studies recruit broader and larger samples of the population (women, minorities) and also recruit specifically for individuals with high levels of early life stress history and statistically control for diagnosis of psychopathology.

The exclusion of patients with psychopathology also limited the number of participants with large numbers of ELS events or with specific types of ELS, such as sexual abuse. On the other hand, even gazing through this narrow window of ELS, we observed important changes in DNA methylation, gene expression, and cortisol response. Use of different measures for ELS, chronic stress, and depressive symptoms allowed us to investigate their contributions as well as control for their effects on each other.

The study design was cross-sectional and based in large part on retrospective self-report, so that some variables of interest, such as ELS, are difficult to verify objectively. Furthermore, as is common in human studies of this kind, our observations are by necessity correlational. Thus, future longitudinal studies in humans, as well as studies based on animal models and *in vitro* processes, will be crucial to complement these results and to further develop mechanistic models of GxE interactions observed here.

A strength of this study is that it classified 5-HTTLPR genotype according to both biallelic and triallelic coding schemes [4,5,52]. However, due to sample size, we lacked the power to analyze each genotype group separately. Future studies with higher sample sizes should investigate these groups separately together with their interactions with early and recent stress measures.

A particularly strong feature of our study was the focus on dynamic changes in gene expression in response to the TSST in a within-subject study design. However, because blood samples were only analyzed for changes between baseline and 105 min after the TSST, it is possible that we did not capture the peak in gene expression. On the other hand, for a subset of the participants, we had gene expression data 45 min after the TSST (*unpublished results*), which suggested a gradual increase in the expression of *SLC6A4* and *NR3C1* from baseline to 105 min after the TSST. In addition, a study by Nater *et al*. [63] reported changes in gene expression in stress- and immune-related pathways as early as 60 min following the TSST, although another study reported continued gene expression changes up to 24 h following stress exposure [64]. Taken together, future studies should measure the expression of candidate genes at multiple time points after the stressor.

Finally, this study had two strong features with regard to its epigenetic analyses: it controlled for global methylation effects [31], and it included the majority of the CpG Units in the *SLC6A4* CpG island. The latter feature is important, because considering only a part of CpG sites maybe misleading, as different CpG sites may vary substantially in terms of their TF binding sites and infrastructure to attract methylation-related proteins [61,65].

One important limitation of this study is that our epigenetic analyses are based on peripheral biomarkers, that is, PBMCs, rather than brain tissue from regions involved in stress processing. Yet, there is growing evidence that DNA methylation patterns may be similar across tissues and species [66-68]. This may also apply to stress-related changes in methylation. For example, in relation to *NR3C1* methylation, the same region associated with ELS was found to be highly methylated in rat [28] and human postmortem hippocampus [30], human leukocytes [32], and cord blood [31]. In addition, there is also recent evidence suggesting effects of peripheral DNA methylation of candidate genes such as *SLC6A4* and catechol-o-methyl transferase on brain activity [42,69]. A multi-pronged approach combining a variety of species *in vivo*, *in vitro*, and postmortem will be required to further elucidate the underlying mechanisms of stress affecting gene expression.

### Future research

In light of the limitations discussed above, future research should be extended to ethnically diverse populations, to women, and to patient populations in order to examine the differential impact of stress and 5-HTTLPR genotype on gene expression, DNA methylation, and HPA response [24,33,70-72].

Future TSST study designs could be further strengthened by including an active control condition. For example, Het and colleagues [73] introduced a placebo version of the TSST, which retained the free speech and arithmetic aspects of the task but removed its stressful features of uncontrollability and social evaluation. Inclusion of an active control condition could further define social stress-related aspects of gene expression and serve as a control condition against possibly spurious correlations. This would also be beneficial to dissociate gene expression changes due to diurnal variation and those induced by stressors like the TSST.

Future work also needs to address causal mechanisms. For example, studies investigating the impact of ELS on *NR3C1* expression across different species and in different tissues (hippocampus to blood) have provided important insights. This multi-pronged approach would also allow us to better understand the neuroanatomical bases of these interactions similar to [74]. In addition, *in vitro* studies investigating the effects of TF binding and methylation at target sites on expression and glucocorticoid response would complement these studies.

Indeed, future studies should focus on mechanistic explanations for differential gene expression, by focusing on the putative role of TF expression and binding *in vitro*. In addition, it would be important to investigate the effect of *SLC6A4* in concert with other genes, such as brain-derived neurotrophic factor (*BDNF*). Previous GxE studies suggested combined effects of 5-HTTLPR, a polymorphism in *BDNF*, and life stress on HPA activity and depression such as [75-77], and a recent review suggested complementary effects of these polymorphisms on the development and chronicity of depression [78]. The association of ELS and *BDNF* methylation is also under investigation [79], making it an ideal gene to investigate its genetic and epigenetic interactions with *SLC6A4*.

## Conclusions

We conclude that individuals respond differently to stress as a function of 5-HTTLPR genotype, both at the level of *SLC6A4* and *NR3C1* gene expression and at the level of gene methylation. Compared to S-group individuals, LL individuals responded with increased *SLC6A4* mRNA levels to the TSST and also show increased global methylation as a function of ELS and chronic stress. Compared to LL individuals, S-group individuals showed reduced *SLC6A4* mRNA levels and increased F3 methylation as a function of ELS; as well as increased F1 methylation as a function of chronic stress and recent depressive symptoms, which correlated positively with *NR3C1* expression. These findings highlight the complex interplay by which an individual’s genotype and type of life stressor may affect DNA methylation and gene expression with relevance to HPA activity, to contribute to individual differences in disease susceptibility or resilience.

## Additional files

Additional file 1:
**Supplementary information.** This file contains more detailed information on exclusion criteria, ELS categorization details, DNA methylation, and qPCR protocols. Additional file 2: Figure S1.
*SLC6A4* (A-B) and *NR3C1* (C-D) gene expression at baseline and in response to the TSST as a function of 5-HTTLPR genotype. There were no significant differences in gene expression by 5-HTTLPR genotype, except for *SLC6A4* expression in response to the TSST. LL individuals increased *SLC6A4* mRNA expression to the TSST whereas S-group individuals remained unchanged; LL individuals had significantly higher *SLC6A4* expression in response to the TSST than did S-group individuals (****P* < .001). Additional file 3: Figure S2.
*NR3C1* expression and cortisol peak response as a function of 5-HTTLPR genotype in the top tertile of *SLC6A4* F1 methylation. For individuals in the lower two tertiles of F1 methylation, there was no correlation between *NR3C1* expression and cortisol response for any of the genotype groups (all *P* values >.05). For individuals in the top tertile, *NR3C1* expression correlated positively with cortisol peak response for S-group participants (*r* (18) = .60, *P* = .006), but not LL participants (*r* (9) = −.22, *P* = .523).

## Abbreviations

5-HT: 5-hydroxytryptamine
5-HTT: 5-hydroxytryptamine transporter
5-HTTLPR: 5-hydroxytryptamine transporter-linked polymorphic region
BDI: Beck Depression Inventory
CTQ: Childhood Trauma Questionnaire
ELS: early life stress
GAPDH: glyceraldehyde 3-phosphate dehydrogenase
GxE: gene-environment interaction
GR: glucocorticoid receptor
HPA: hypothalamic-pituitary-adrenal axis
HPRT1: hypoxanthine phosphoribosyltransferase
LINE-1: Long Interspersed Nuclear Element-1
NGFI-A: nerve growth factor-inducible protein A
NR3C1: nuclear receptor subfamily 3 group C member 1
PBMC: peripheral blood mononuclear cell
SLC6A4: solute carrier family 6 member 4
SLE: stressful life event
SNP: single nucleotide polymorphism
TICS: Trier Inventory of Chronic Stress
TF: transcription factor
TSST: Trier Social Stress Test
UNG: uracil-N-glycosylase
