# Supplementary material for: Influence of life stress, 5-HTTLPR genotype, and SLC6A4 methylation on gene expression and stress response in healthy Caucasian males
Source: Biol Mood Anxiety Disord. 2015 May 14;5:2. doi: 10.1186/s13587-015-0017-x (PMC4438516; doi:10.1186/s13587-015-0017-x)
Supplement: Additional file 1: — Supplementary information. This file contains more detailed information on exclusion criteria, ELS categorization details, DNA methylation, and qPCR protocols. [file 13587_2015_17_MOESM1_ESM.docx]

**Supplementary Information**

**Exclusion criteria for participants**

Individuals who smoked, reported to be under immense stress currently, had habitual substance and/or alcohol abuse in the last 6 months, had high (>30) or low (<18) BMI, thyroid disease, diabetes, or previous diagnosis of mental health problems or use of any kind of mood- or HPA-altering medication were ineligible due to the effects of these factors on HPA reactivity [reviewed in [1](#_ENREF_1), [2](#_ENREF_2)]. Finally, individuals were asked about their fear of having their blood drawn on a scale from 1 (no fear) to 7 (extreme fear). Only individuals with scores lower than 4 were recruited.

**ELS categorization details**

In addition to using CTQ total scores as a continuous measure, participants were also grouped in two ways. First of all, CTQ total scores were divided into tertiles, creating the low, moderate and high ELS groups. Secondly, previously used cut off scores for trauma [[3](#_ENREF_3)] for each subscale was taken (physical abuse = 10, emotional abuse = 13, sexual abuse = 8, physical neglect = 10 and emotional neglect = 15) to divide participants into ‘ELS’ or ‘no-ELS’ groups. Those scoring higher than the cut off values in one or more of the subscales were placed in the ‘ELS’ group (*n* = 18) and those scoring lower than the cut off scores in all the subscales were placed in the ‘no-ELS’ group (*n* = 87). In the ‘ELS’ group, 6 participants were LL homozygotes and 12 were S-carriers. In the ‘no-ELS’ group, 29 participants were LL homozygotes and 58 were S-carriers.

**DNA methylation protocol details**

**LINE-1 methylation analysis.** The region analyzed included 4 CpG sites and average DNA methylation of these sites was used as the global methylation level. In addition to the 0% and 100% methylation controls, samples with no template and no sequencing primer were used as negative controls and all PCR products were run on agarose gels for verification of the amplification prior to pyrosequencing. The pyrosequencing reactions were prepared with 20 µl of PCR product according to manufacturer’s protocol and assayed in duplicates in the PyroMark Q96 MD system and methylation levels were obtained by the PyroMark software (Qiagen, CA).

**SLC6A4 CpG island DNA methylation analysis.** In Sequenom Epityper MassArray system (San Diego, CA) DNA fragments of interest are bisulfite-converted, PCR-amplified and then cleaved into smaller units called *CpG Units* that contain one or more CpG sites [[4](#_ENREF_4)]. PCRs were carried out with 2 µl bisulfite treated DNA and 160 nM from each primer. Primers for the first and second amplicons were Amplicon1: 5’GGGTTTTTATATGGTTTGATTTTTAGA and 5’CACCTACTCCTTTATACAACCTCCC, Amplicon 2: 5’GGTTATTTAGAGATTAGATTATGTGAGGG and 5’CTACAACAATAAACAAAAAAACCCC, respectively, with the standard Sequenom tails. The successful amplification of PCR products were checked on agarose gels and samples were aliquoted in triplicates into 384-well plates and shipped on dry ice to the Genomics Facility of Albert Einstein School of Medicine, New York, where the *in vitro* RNA transcription and base specific cleavage was performed and the samples were analyzed through MALDI-TOF mass spectrometry [[4](#_ENREF_4)].

Out of 37 CpG Units analyzed, five of them could not be analyzed via the mass spectrometry technique due to their high or low mass. In addition, two duplicate CpG Units that have the same mass and thus yield identical results were excluded. Finally, one CpG Unit was excluded from analysis due to highly inconsistent results between replicates in more than one third of the participants and one CpG Unit was excluded due to yielding results in only less than one third of the participants.

**qPCR protocol details**

The primers used for reference genes and genes of interest are shown in Supplementary Table 1. For all reactions, melting curve analysis was conducted to ensure successful amplification. Samples with no cDNA and no reverse transcriptase were used as negative controls. In addition, in order to control for variability between runs, the same cDNA sample was analyzed in all plates (inter-plate CV < 5%).

In order to determine the best reference genes in PBMCs, expression of 6 candidate reference genes were tested (*B2M, GAPDH, HPRT1, PPIA, SDHA, YWHAZ*) in 5 participants’ samples at baseline and in response to the TSST. GenEx software (MultiD Analyses AB, Göteborg, Sweden) was used which featured built-in reference gene selection tools [[Genorm by 5](#_ENREF_5), [NormFinder by 6](#_ENREF_6)]. This analysis identified *HPRT1* (hypoxanthine phosphoribosyltransferase 1) and *GAPDH* (glyceraldehyde 3-phosphate dehydrogenase) as the best reference genes in PBMCs. Amplification efficiencies of the target and reference genes were determined from standard curves and were similar. C_T_ values of these genes were used to normalize *SLC6A4* and *NR3C1* expression in all samples. *NR3C1* primers were specific to GR-α since the other two forms (GR-β and GR-P) were beyond detection levels in PBMCs (C_T_ > 40). Previous research reported positive correlations between these three forms in PBMCs [[7](#_ENREF_7)] and associated particularly GR-α with changes in HPA activity and depression [[8](#_ENREF_8)]. Baseline gene expression was calculated as log-transformed delta-C_T_ values. There were three outliers in *SLC6A4* expression and one outlier for *NR3C1* expression that were excluded from analyses (*z*-scores > 3). Primer sequences for the reference genes and genes of interest are given in Supplementary Table 2.

| **Supplementary Table 1. Correlations between CTQ total and subscale scores.** | | | | | | |
| --- | --- | --- | --- | --- | --- | --- |
| Measure | 1 | 2 | 3 | 4 | 5 |  |
| 1. CTQ Total | − |  |  |  |  |  |
| 2. CTQ emotional abuse | .84^**^ | − |  |  |  |  |
| 3. CTQ emotional neglect | .86^**^ | .54^**^ | − |  |  |  |
| 4. CTQ physical abuse | .71^**^ | .63^**^ | .39^**^ | − |  |  |
| 5. CTQ physical neglect | .67^**^ | .36^**^ | .53^**^ | .31^*^ | − |  |
| *Note*. ^*^ *p* = .001; ^**^ *p* < .001. CTQ sexual abuse was not included because of the presence of only two individuals with scores higher than 5 (possible scores range 5-25). | | | | | | |

| **Supplementary Table 2. qPCR primer sequences for reference and target genes.** | |
| --- | --- |
| Gene | 5’ to 3’ primer sequence |
| *GAPDH* | F: cgctgagtacgtcgtggag  R: gcagagatgatgacccttttg |
|  |  |
| *HPRT1* | F: TGACACTGGCAAAACAATGCA  R: GGTCCTTTTCACCAGCAAGCT |
|  |  |
| *NR3C1* | F: GAAGGAAACTCCAGCCAGAACTG  R: GATGATTTCAGCTAACATCTC |
|  |  |
| *SLC6A4* | F: TGTCTGAGGTGGCCAAAGA  R: GTTGGCTATCGCTTCTGCAT |
|  |  |

| **Supplementary Table 3. Correlations between ELS and *SLC6A4* methylation as a function of 5-HTTLPR genotype.** | | |
| --- | --- | --- |
| Measure | S-group | LL |
| F1 methylation | -.12 | -.26 |
| F2 methylation | .23 | -.06 |
| F3 methylation | **.30^*^** | -.28 |
| *Note*. Partial correlations controlling for age and *LINE-1* methylation. ^*^ *p* < .05 | | |


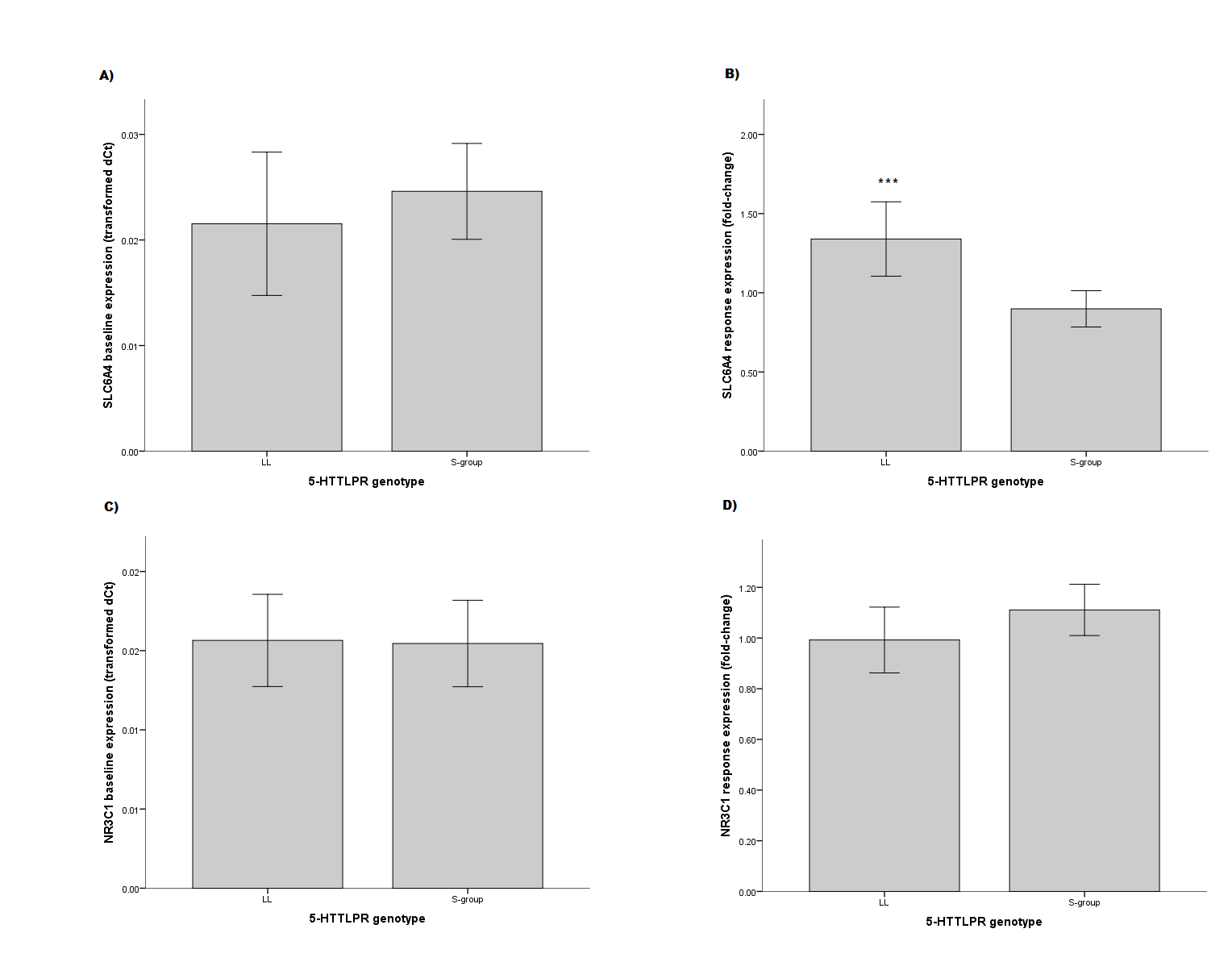


**Supplementary Figure 1. *SLC6A4* (A-B) and *NR3C1* (C-D) gene expression at baseline and in response to the TSST as a function of 5-HTTLPR genotype.** There were no significant differences in gene expression by 5-HTTLPR genotype, except for *SLC6A4* expression in response to the TSST. LL individuals increased *SLC6A4* mRNA expression to the TSST whereas S-group individuals remained unchanged; LL individuals had significantly higher *SLC6A4* expression in response to the TSST than did S-group individuals (*** *p* < .001).


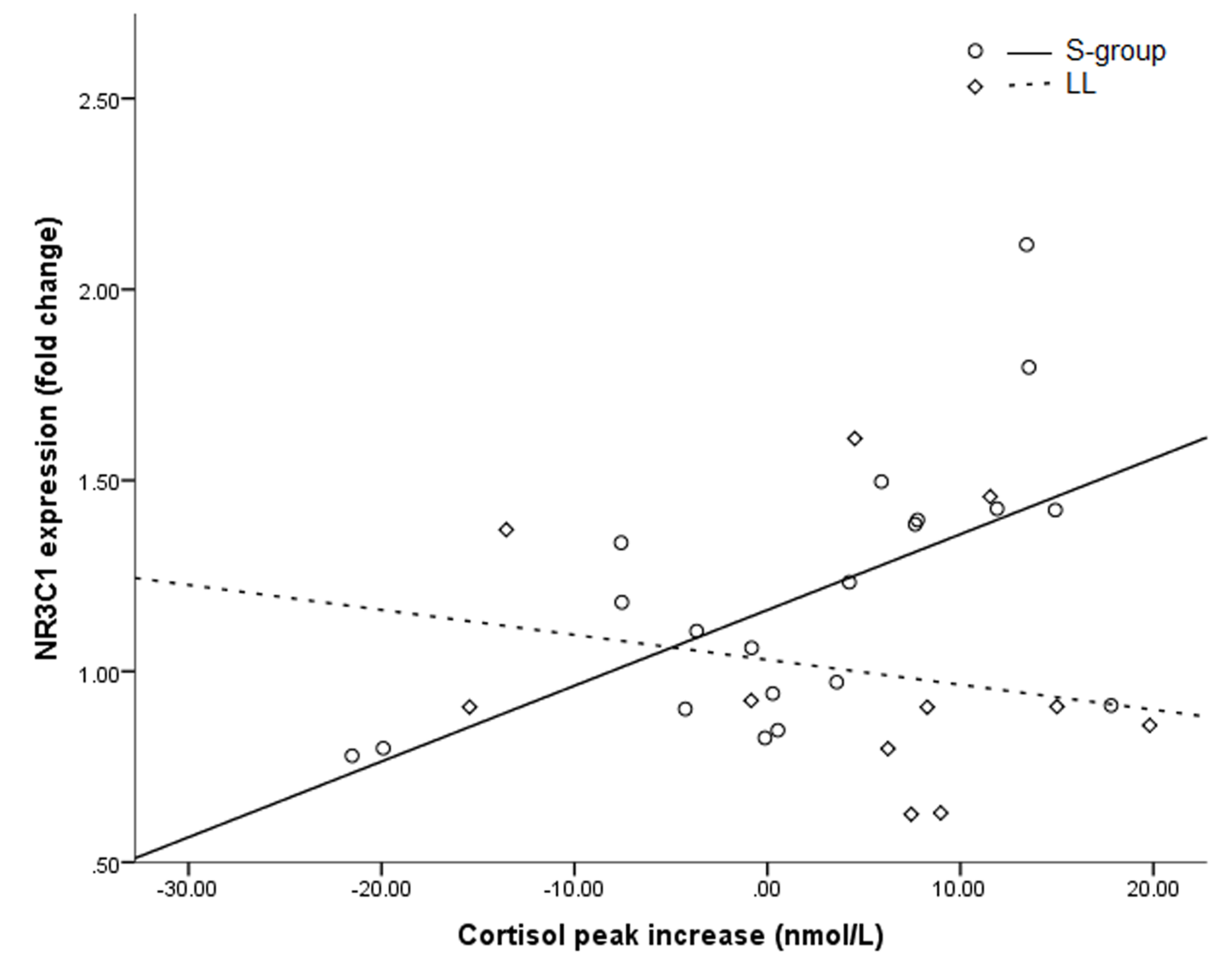


**Supplementary Figure 2.** ***NR3C1* expression and cortisol peak response as a function of 5-HTTLPR genotype in the top tertile of *SLC6A4* F1 methylation.** For individuals in the lower two tertiles of F1 methylation, there was no correlation between *NR3C1* expression and cortisol response for any of the genotype groups (all p-values > .05). For individuals in the top tertile, *NR3C1* expression correlated positively with cortisol peak response for S-group participants (r (18) = .60, p = .006), but not LL participants (r (9) = -.22, p = .523).

**Supplementary references**

1. Kudielka B, Hellhammer D, Wüst S: **Why do we respond so differently? Reviewing determinants of human salivary cortisol responses to challenge.** *Psychoneuroendocrinology* 2009, **34:**2-18.

2. Foley P, Kirschbaum C: **Human hypothalamus–pituitary–adrenal axis responses to acute psychosocial stress in laboratory settings.** *Neuroscience & Biobehavioral Reviews* 2010, **35:**91-96.

3. Wankerl M, Miller R, Kirschbaum C, Hennig J, Stalder T, Alexander N: **Effects of genetic and early environmental risk factors for depression on serotonin transporter expression and methylation profiles.** *Translational Psychiatry* 2014, **4:**e402.

4. Ehrich M, Nelson MR, Stanssens P, Zabeau M, Liloglou T, Xinarianos G, Cantor CR, Field JK, Van Den Boom D: **Quantitative high-throughput analysis of DNA methylation patterns by base-specific cleavage and mass spectrometry.** *Proceedings of the National Academy of Sciences of the United States of America* 2005, **102:**15785.

5. Vandesompele J, De Preter K, Pattyn F, Poppe B, Van Roy N, De Paepe A, Speleman F: **Accurate normalization of real-time quantitative RT-PCR data by geometric averaging of multiple internal control genes.** *Genome Biology* 2002, **3:**research0034.

6. Andersen CL, Jensen JL, Ørntoft TF: **Normalization of real-time quantitative reverse transcription-PCR data: a model-based variance estimation approach to identify genes suited for normalization, applied to bladder and colon cancer data sets.** *Cancer research* 2004, **64:**5245.

7. Hagendorf A, Koper JW, de Jong FH, Brinkmann AO, Lamberts SW, Feelders RA: **Expression of the human glucocorticoid receptor splice variants α, β, and P in peripheral blood mononuclear leukocytes in healthy controls and in patients with hyper-and hypocortisolism.** *The Journal of Clinical Endocrinology & Metabolism* 2005, **90:**6237-6243.

8. Matsubara T, Funato H, Kobayashi A, Nobumoto M, Watanabe Y: **Reduced glucocorticoid receptor α expression in mood disorder patients and first-degree relatives.** *Biological psychiatry* 2006, **59:**689-695.
