# Supplementary figures and images for: Influence of life stress, 5-HTTLPR genotype, and SLC6A4 methylation on gene expression and stress response in healthy Caucasian males
Source: Biol Mood Anxiety Disord. 2015 May 14;5:2. doi: 10.1186/s13587-015-0017-x (PMC4438516; doi:10.1186/s13587-015-0017-x)

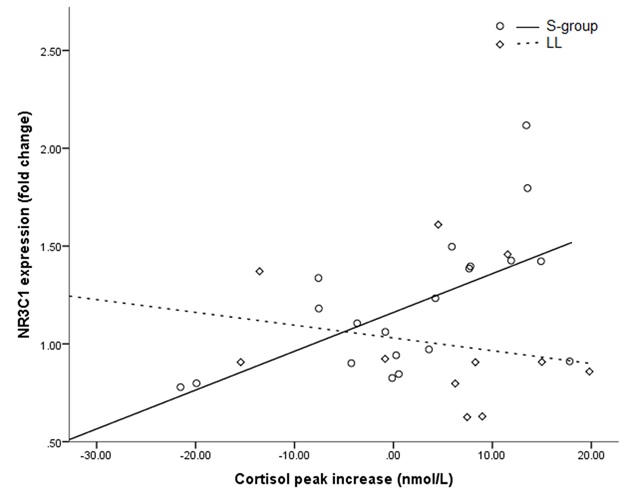

Supplement: Additional file 3: Figure S2. — NR3C1 expression and cortisol peak response as a function of 5-HTTLPR genotype in the top tertile of SLC6A4 F1 methylation. For individuals in the lower two tertiles of F1 methylation, there was no correlation between NR3C1 expression and cortisol response for any of the genotype groups (all P values >.05). For individuals in the top tertile, NR3C1 expression correlated positively with cortisol peak response for S-group participants (r (18) = .60, P = .006), but not LL participants (r (9) = −.22, P = .523). [file 13587_2015_17_MOESM3_ESM.jpeg]
